# Glycosaminoglycan Modification of Decorin Depends on MMP14 Activity and Regulates Collagen Assembly

**DOI:** 10.3390/cells9122646

**Published:** 2020-12-09

**Authors:** Alexes C. Daquinag, Zhanguo Gao, Cale Fussell, Kai Sun, Mikhail G. Kolonin

**Affiliations:** The Brown Foundation Institute of Molecular Medicine, University of Texas Health Science Center, 1825 Pressler St. 430E, Houston, TX 77030, USA; Alexes.Daquinag@uth.tmc.edu (A.C.D.); Zhanguo.Gao@uth.tmc.edu (Z.G.); Cale.G.Fussell@uth.tmc.edu (C.F.); Kai.Sun@uth.tmc.edu (K.S.)

**Keywords:** decorin, collagen, glycosaminoglycan, matrix metalloprotease, adipose, muscle

## Abstract

Proper processing of collagens COL1 and COL6 is required for normal function of adipose tissue and skeletal muscle. Proteoglycan decorin (DCN) regulates collagen fiber formation. The amino-terminus of DCN is modified with an O-linked glycosaminoglycan (GAG), the function of which has remained unclear. Previously, non-glycanated DCN (ngDCN) was identified as a marker of adipose stromal cells. Here, we identify MMP14 as the metalloprotease that cleaves DCN to generate ngDCN. We demonstrate that mice ubiquitously lacking DCN GAG (ngDCN mice) have reduced matrix rigidity, enlarged adipocytes, fragile skin, as well as skeletal muscle hypotrophy, fibrosis, and dysfunction. Our results indicate that DCN deglycanation results in reduced intracellular DCN—collagen binding and increased production of truncated COL6 chains, leading to aberrant procollagen processing and extracellular localization. This study reveals that the GAG of DCN functions to regulate collagen assembly in adipose tissue and skeletal muscle and uncovers a new mechanism of matrix dysfunction in obesity and aging.

## 1. Introduction

Normal function of cells relies on the extracellular matrix (ECM), a complex and dynamic scaffold that enables structural support and mediates signal transduction. Alterations in ECM structure and function result in abnormal tissue remodeling, which underlies pathogenesis of various diseases [1]. Collagens comprise the integral family of ECM proteins. All collagen molecules are composed of three α chains. These chains assemble into striated fibrils in fibrillar collagens. Collagens type I (COL1) and type VI (COL6) are the most abundant and are essential for normal ECM function [2]. This is evident from congenital defects caused by mutations in these proteins. Mutations in *COL1* gene cause osteogenesis imperfecta, as well as loose joints, stretchy skin, and abnormal scarring [3]. Mutations in *COL6* cause several types of myopathy and congenital muscular dystrophy, as well as skin defects [4,5]. On the other hand, dysregulated production of COL1 and COL6 underlies fibrosis, aggravating various chronic pathological conditions. Examples include obesity, resulting from adipose tissue (AT) overgrowth and inflammation, as well as cancer. Pathogenesis in these conditions often implicates aberrantly assembled ECM molecules that undergo abnormal proteolysis and other post-translational modifications [6,7]. Collagenases, the metalloproteinases proteolytically processing collagens, are important modulators of collagen homeostasis and ECM properties [8].

Interaction of ECM with cells is mediated by matricellular proteins, which modulate cell function by linking collagens with proteases, cell-surface receptors, and their ligands, such as growth factors [9,10,11]. One of the collagen-binding matricellular proteins is decorin, a small leucine-rich proteoglycan expressed in most solid tissues [12]. By modulating ECM–cell interactions, DCN plays a role in wound healing and angiogenesis [13]. DCN regulates assembly of the collagen fibrils and bioactivity of the matrix-associated growth factors [12,14]. The key function of DCN is the regulation of collagen fibril diameter and orientation of the collagen fibrils, which thereby controls cell adhesion, migration, and differentiation [12,15]. During fibrillogenesis, DCN increases the modulus and tensile strength of the resulting collagen matrix due to the reduced aggregation of fibrils into bundles [16,17]. The importance of DCN for this process is evident from the phenotype of DCN-null mice, which have loose and fragile connective tissue due to irregular collagen fibers [18].

The amino-terminus of core DCN is normally modified with a large O-linked glycosaminoglycan (GAG), chondroitin, or dermatan sulfate chain [19,20]. Diminished GAG modification of DCN is a hallmark of gerodermia osteodysplastica, a disease characterized by skin laxity and early-onset osteoporosis [21]. Consistent with this, a reduction in DCN glycanation is observed in skin during aging, as well as in patients with Ehlers–Danlos syndrome displaying a skin-fragility phenotype [22]. Potentially explaining these phenotypes, the GAG chain of DCN has been shown to play a role in collagen fibril formation at the early stages of fibrillogenesis [17]. However, no abnormalities in development or wound healing were observed in a mouse strain with DCN lacking the GAG chain of DCN [23]. Therefore, the debated function of DCN glycan and the pathophysiological role of deglycanated DCN need to be further elucidated.

**Figure 1:**
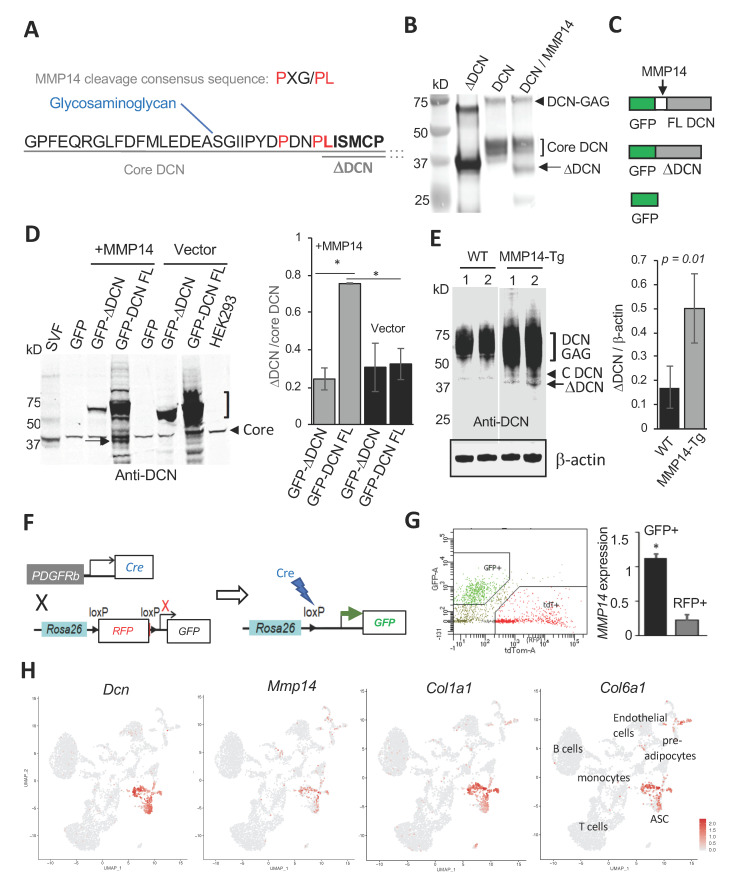
MMP14 is a protease generating a non-glycanated DCN isoform. (**A**) N-terminal sequence of mouse core DCN with downstream amino acid sequence abbreviated (…), showing serine-attached GAG and ΔDCN starting at leucine-45. The consensus MMP14 recognition motif is aligned above, showing matches (red). (**B**) ΔDCN (purified from bacteria) and full length (FL) DCN (purified from NS0 cells) were incubated with (+) or without (−) MMP14 for 30 min, resolved by 4–20% SDS-PAGE and subjected to anti-DCN immunoblotting showing cleavage of core DCN (bracket) and generation of ΔDCN (arrow). Arrowhead: glycanated DCN. (**C**) Recombinant GFP constructs expressed in HEK293 cells to test cleavage by MMP14. (**D**) Anti-DCN immunoblotting of extracts from HEK293 co-transfected with MMP14 and constructs shown in (**C**) reveals that ΔDCN (arrow) is induced by MMP14 expression. Note that ΔDCN-GFP is not cleaved. SVF (from mouse WAT): positive control for ΔDCN. HEK293 (non-transfected): negative control for ΔDCN. Bracket: glycanated DCN. Endogenous core DCN detected in all cells is used for normalization (graph). N = 3 independent transfections; * *p* < 0.01 (Student’s *t*-test). (**E**) Anti-DCN immunoblotting of extracts from WAT demonstrates higher ΔDCN (arrow) production in MMP14-overexpressing mice, compared to WT mice (N = 5 mice per group), for which only core glycanated (DCN-GAG) and core DCN (arrowhead) is detected. 1 and 2: separate littermates. Anti-β-actin immunoblotting: loading control used for normalization (graph). (**F**) A scheme for lineage tracing in the progeny of the cross between *Pdgfrb-Cre* and *mTmG* mice. Upon Cre expression and LoxP site recombination in *Pdgfrb*+ cells mT (membrane Tomato) is deleted and RFP is indelibly replaced by membrane GFP expression. (**G**) Isolation of GFP+ and RFP+ cells by FACS from WAT of *Pdgfrb-Cre*; *mTmG* mice and their RT-PCR analysis, which reveals high *MMP14* mRNA expression in ASC identified as mG+ cells (*Pdgfrb*+ lineage). N = 5 independent samples. * *p* < 0.05 (Student’s *t*-test) compared to mT+ cells. The RT-PCR data are normalized to *18S RNA*. (**H**) ScRNA-seq analysis of gene expression in Uniform Manifold Approximation and Projection (UMAP) plots showing clusters of cells identified. Note co-expression of *Dcn*, *Mmp14*, *Col1a1*, and *Col6a1* in ASC.

DCN is highly expressed in white adipose tissue (WAT), in particular in obesity [24]. We previously discovered a non-glycanated isoform of DCN in WAT. Expansion of WAT in obesity and its maintenance relies on adipose progenitor cells (APC) [25,26,27]. These cells are a sub-population of adipose stromal cells (ASC) similar to mesenchymal stromal cells (MSC), which, like in other organs, also serve as vasculature-supporting ECM-producing cells [28,29]. Through a screen of a combinatorial library, we have previously identified a peptide, WAT7 [30], which homes in on the adipocyte progenitor subpopulation of ASC that expresses platelet-derived growth factor receptor beta (PDGFRβ) [31,32]. We identified that WAT7 binds a proteolytic cleavage fragment of core DCN, termed delta-decorin (ΔDCN), which lacks the N-terminus containing the serine serving as a GAG attachment site (Figure 1A). This non-glycanated DCN isoform is expressed on the ASC surface, which has been used to target mouse and human ASC [30,31]. However, the function of the ΔDCN as well as the protease cleaving DCN to generate ΔDCN in WAT has not been previously investigated.

Here, we show that MT1-MMP (also known as MMP14), a collagenase highly expressed in AT [33] and further induced in obesity [34], is co-expressed with DCN in ASC and cleaves DCN to generate ΔDCN. We demonstrate that mice lacking DCN GAG and constitutively expressing non-glycanated DCN (ngDCN mice) display skin, WAT, and skeletal muscle defects akin to those observed for fibrillar collagen mutations. Analysis of collagens I and VI in mouse tissues and cells indicated that ngDCN binding to collagen chains increases their lysosome-targeted proteolysis and interferes with normal chain assembly. Our data provide new evidence that the GAG of DCN is important for normal collagen production and suggest that increased DCN deglycanation could contribute to matrix stiffness loss, adipocyte hyperplasia, and sarcopenia in obesity and aging.

## 2. Materials and Methods

### 2.1. Cell Culture

Cells were grown in DMEM/10% FBS. Primary ASC were isolated from male mice and embryonic fibroblasts (MEFs) were isolated from mixed male and female mice as described [30,35,36,37]: minced WAT (for ASC isolation) or day 14 mouse embryos (for MEF isolation) were digested in 0.5 mg/mL collagenase type I (Worthington Biochemical) and 2.5 mg/mL of dispase (Roche, Indianapolis, IN, USA, Cat. # 04942078001) solution under gentle agitation for 1 h at 37 °C and centrifuged at 400× *g* for 5 min to isolate the stromal-vascular fraction (SVF) pellet. For GFP fusion expression, cDNA fragments coding for full-length mouse core DCN and ΔDCN isoforms were cloned into the pLVX-AcGFP-C1 vector as described [30]. MMP14-expression constructs were described previously [7]. HEK293 cells were grown into 80% confluence before co-transfecting with GFP fusion expression plasmids and MMP14-harboring or control pcDNA3.1 vector. A total of 48 h after transfection, HEK293 cells were harvested and subjected to SDS-PAGE and Western blotting. For adipogenesis induction, preadipocytes from wild-type (WT) or ngDCN mice grown to confluence were cultured in medium containing 1.7 µM insulin/0.5 mM IBMX, 1 μM dexamethasone/0.2 mM indomethacin for 3 days, and 1.7 µM insulin afterwards.

### 2.2. Binding Assays and Immunoblotting

Isolation of proteins from cells and tissues was performed as described [37]. NS0-expressed murine DCN was from R&D Systems, Minneapolis, MN, USA, #1060-DE. Recombinant Human MMP-14/MT1-MMP Protein (918-MP-010) was from R&D Systems. His_6_-tagged ΔDCN cloned into the pET28a vector with _6_His at the N-terminus was purified with the His-Tag kit (Novagen, Burlington, MA, USA) as described [30]. For Western blotting, lysates were resolved on SDS-PAGE, blotted onto an Immobilon-FL membrane (Millipore, Burlington, MA, USA), blocked with Odyssey blocking buffer (LI-COR), and probed (in PBS/0.05% Triton X-100) with antibodies as follows: goat anti-mouse DCN from R&D Systems (#BAF1060) at 1:1000; rabbit monoclonal anti-Col6A1 (Abcam, Cambridge, MA, USA; ab182744) at 1:1000; rabbit polyclonal anti-Col6A1 (Abcam ab6588) at 1:1000; rabbit polyclonal anti-Co1A1 (Abcam #34710) at 1:1000; Rabbit anti-endotrophin antibody [38] at 1:1000; Rabbit anti-GFP (Abcam ab290) at 1:1000; and anti-β-Actin (Abcam, Cat. # ab8226, 1:5000). The signal was detected using the Odyssey CLx imaging system (LI-COR, Lincoln, NE, USA).

### 2.3. Fluorescence-Activated Cell Sorting (FACS) and Expression Analyses

SVF mT and mG cells were separated into populations by FACSAria/FacsDiva software (BD Biosciences, San Jose, CA, USA). RNA was extracted using the Trizol Reagent (Life Technologies, Waltham, MA, USA; Cat. # 15596018). Complementary DNAs were generated using the High Capacity cDNA Reverse Transcription Kit (Applied Biosystems, Foster City, CA; Cat. # 4368814). PCR reactions were performed on a CFX96™ Real-Time System C1000 Touch thermal cycler (Bio-Rad, Hercules, CA, USA) using Q-PCR Master Mix (Gendepot, Katy, TX, USA; Cat. # Q5600-005). Expression of mouse genes was normalized to *18S RNA*. The Sybr green primers were as follows:

*18S RNA*: 5′-AAGTCCCTGCCCTTTGTACACA-3′, 5′-GATCCGAGGGCCTCACTAAAC-3′;

*MMP14*: 5′-TGGATACCCAATGCCCATTG-3′, 5′-CTTCGTCAAACACCCAGTGCTT-3′.

### 2.4. Mouse Experiments

Mice were housed in the animal facility with a 12-h light/dark cycle and constant temperature (22–24 °C) unless indicated otherwise. Animals had free access to water and diet. All animal experimentations complied with protocols approved by the UTHealth Animal Care and Use Committee. The crosses between reporter *mT/mG* mice from Jackson Laboratories (Stock No. 007676) and *Pdgfrβ-Cre* mice [39] were characterized and used as described [36]. Generation and doxycycline induction of MMP14 overexpression in transgenic mice was described previously [7]. In most experiments, age-matched male ngDCN and WT (control) littermates were used; both males and females were used for skin analysis. For obesity induction, mice were fed 58 kcal% (fat) HFD (Research Diets, New Brunswick, NJ, USA; D12331). Body composition was measured by EchoMRI-100T (Echo Medical Systems, Houston, TX, USA). Exer-3/6 Treadmill 1055-SDRMAI-D60 equipped with Shock detection/auto-calibration was used to measure fatigue resistance. Work performed was calculated as mouse weight (kg) × speed (m/min) × time (min) × incline (degree) × 9.8 m/sec squared. Indirect calorimetry studies and food intake measurements (over a 3-day time course) were performed with an OXYMAX (Columbus Instruments, Columbus, OH, USA) Comprehensive Lab Animal Monitoring System (CLAMS) as described [35,36]. Core body temperature was determined in the rectum at 2.5 cm deep using a MicroTherma 2K High Precision Type K Thermocouple Meter (THS-221-092, ThermoWorks, American Fork, UT, USA) with a RET-3 probe (Braintree Scientific, Braintree, MA, USA). Cold tolerance was measured upon placing mice into an environmental chamber (IS33SD, Powers Scientific) as described [36]. Tumor cells were subcutaneously grafted onto the upper flank as described [40].

### 2.5. Single Cell RNA Sequencing

Single-cell capture (~3000 cells/sample) and library construction were performed with the Chromium Single Cell 3′ Reagent Kit v3.1. Barcoded single-cell gel beads were loaded onto a Chromium Next GEM ChipG (PN-1000120). After running on a 10× Chromium Single Cell Controller, gel beads-in-emulsion (GEMs) were generated. The barcoded and full-length cDNAs were produced after incubation of the GEMs and amplified via PCR. The library was qualified by an Agilent Bioanalyzer 2100 and quantified by real-time PCR on QuantStudio3. Sequencing was done with an Illumina NextSeq 550 System using High Output Kit v2.5 (50,000 reads per cell). The Cell Ranger™ Single Cell Software Suite v.3.1.0 was used to perform the bioinformatic analysis. The reads were aligned to the mouse transcriptome reference (mm10, Ensembl 93) with STAR [41]. Raw read count tables were analyzed using the Seurat (v3.1.1) pipeline on the R platform (3.5.2). FindVariableGenes was used to calculate the principal components. Cell clusters were identified using the Shared Nearest Neighbor algorithm with a resolution parameter of 0.8. UMAPs were based on the first 10 principal components and feature plots were displayed with the log (raw read count + 1) of gene/cell on UMAP.

### 2.6. Tissue Analysis

Formalin-fixed, paraffin-embedded tissue sections were analyzed by Masson’s trichrome, hematoxylin/eosin (H&E) staining or by immunofluorescence (IF) as described [30,35,36,37]. Collagen deposition was quantified using the Sirius Red/Fast Green kit (Chondrex, Woodinville, WA, USA; #9046). Cultured cells were fixed with 4% paraformaldehyde. Upon blocking, the following primary antibodies (4 °C, 12 h) and secondary antibodies (RT, 1 h) diluted in phosphate-buffered saline (PBS) with 0.05% Tween 20 were used: goat anti-DCN (R&D Systems BAF1060 at 1:200); rabbit anti-Col6A1 (Abcam ab182744 at 1:75; and ab6588 at 1:100); rabbit anti-Co1A1 (Abcam ab34710 at 1:75); Donkey Alexa 488-conjugated (1:200) IgG from Invitrogen; and Cy3-conjugated (1:300) IgG from Jackson ImmunoResearch, West Grove, PA, USA. Nuclei were stained with Hoechst 33258 (Invitrogen Waltham, MA, USA; H3569). IF images were acquired with a Carl Zeiss upright Apotome Axio Imager Z1/ZEN2 Core Imaging software. Quantification was done using NIH ImageJ software by counts in 10 separate 10× fields. Amira 5.4 software (VSG) was used for data capture and analysis. Confocal images were acquired with TCS SP5/LAS AF software (Leica, Buffalo, Grove, IL, USA).

### 2.7. Atomic Force Microscopy

Tissue stiffness was measured as described previously [42] by the UTHealth AFM Core Facility. Skin specimens were snap-frozen in OCT and cryosectioned (50 µm thick) on polylysine-coated microscope slides. Samples were brought to room temperature, rinsed with PBS to remove OCT, air-dried, and re-hydrated for 1 h before use. Tissue samples were kept at RT in PBS during AFM measurements by using a BioScope IITM atomic force microscope (Bruker Corporation; Santa Barbara, CA, USA) integrated with a Nikon TE2000-E inverted optical microscope to facilitate area selection. Force curves from 10 randomly chosen points along the longitude of the dermis and hypodermis were registered using AFM non-conductive silicon nitride DNP-S cantilevers (fo = 40–75 kHz, k = 0.32 N/m, and ROC = 10 nm) purchased from Bruker Corporation (Santa Barbara, CA, USA). Indentation curves were probed using a ramp size of 5 µm and scan rate of 0.5 Hz, with a maximum force load of 10 nN. Each cantilever was calibrated for its laser sensitivity using the thermal oscillation method prior to each experiment. Young’s modulus was calculated by fitting to a standard Sneddon model for a triangular indenter (radius of 10 nm) and a Poisson’s ratio of 0.5 (constant for tissue samples). A minimum of 30 force measurements were taken in each sample. AFM data analysis was performed with NanoScope Analysis software (version 1.50, copyright 2015 Bruker Corporation) to estimate Young’s modulus.

### 2.8. Statistical Analysis

Microsoft Excel was used to graph the data as the mean ± SEM and to calculate the *p*-values using homoscedastic Student’s *t*-tests. A *p* < 0.05 was considered significant. All experiments were repeated at least twice with similar results.

## 3. Results

### 3.1. MMP14 Expressed by ASC Cleaves DCN and Generates ngDCN

MMP14, a collagenase active in remodeling WAT [33], has been previously reported to cleave DCN [43]. However, the precise cleavage site(s) in DCN has not been characterized. Analysis of the DCN sequence in the area of the ΔDCN N-terminus identified a sequence similar to the MMP14 recognition consensus motif (P-X-G/P-L) [44], with 3/4 amino acids matching (Figure 1A). To test for cleavage at that site, we incubated C-terminally His_10_-tagged full-length (FL) mouse DCN (purified from NS0 cells) with (or without) MMP14 in vitro. The products were resolved on a denaturing polyacrylamide gel along with ΔDCN containing a His_6_-tag and a 22 amino acid linker at the N-terminus [30]. Immunoblotting with an anti-DCN antibody revealed that FL DCN was proteolyzed by MMP14, resulting in a band of a size predicted for ΔDCN (Figure 1B). Mass spectrometry and immunoblotting with an anti-His tag antibody (Figure S1A) confirmed the band as a C-terminal fragment starting at the MMP14 consensus motif. Because the 40 kDa core DCN band is reduced by MMP14, our data indicate that GAG is not required for cleavage by MMP14.

To test if MMP14 cleaves DCN in cells, we generated plasmids for expression of recombinant mouse DCN or ΔDCN in frame with GFP at the N-terminus (Figure 1C). These plasmids were transfected into HEK293 cells. GFP fluorescence confirmed expression of all constructs and revealed cell surface ΔDCN localization (Figure S1B). The cells were co-transfected with a plasmid expressing MMP14 or an empty vector as a negative control. The products were resolved on a denaturing gel, with a membrane extract from mouse SVF, expressing ΔDCN, used as a size marker. Cleavage by MMP14 was analyzed by anti-DCN immunoblotting. Because ΔDCN lacks the MMP14 cleavage site, we predicted that GFP fusion with full-length DCN, but not with ΔDCN, would be cleaved. As expected, comparable levels of ~40 kDa endogenous core DCN were observed for all lanes, while glycanation was prominent only for full-length DCN-GFP (Figure 1D). The band matching the size of ΔDCN was detected for cells expressing full-length DCN-GFP but not for ΔDCN-GFP fusion (Figure 1D). Importantly, this band was at the background level without ectopic MMP14 expression (Figure 1D). The result was verified by anti-GFP immunoblotting: MMP14 expression induced the production of free GFP migrating at ~27 kDa preferentially in cells expressing full-length DCN-GFP (Figure S1C). MMP14 small molecule inhibitor NSC405020 and a blocking antibody 3A2 [7] added to MMP14 co-expressing cells suppressed the release of ΔDCN from the full-length DCN-GFP fusion, further reinforcing our conclusions (Figure S1D).

To further confirm that MMP14 cleaves DCN in vivo, we used mice ectopically expressing MMP14 in WAT, which our group described recently [7]. By crossing a strain with tetracycline response element (TRE) upstream of the MMP14 gene and a strain expressing reverse tetracycline-controlled transactivator (rtTA) under the control of adiponectin promoter [45,46], we generated progeny overexpressing MMP14 in the adipocyte lineage upon doxycycline treatment. Littermates lacking the rtTA-expressing transgene were used as a WT control. Protein extracts from WAT were resolved on a denaturing gel and subjected to anti-DCN immunoblotting. Mainly glycanated and core DCN were detected in WAT lysates from WT mice (Figure 1E). In contrast, ΔDCN production was significantly increased in WAT lysates of mice overexpressing MMP14 (Figure 1E). These in vitro and in vivo studies demonstrate that MMP14 cleaves full-length DCN to generate ΔDCN.

Next, we sought to investigate why ASCs are the cell population selectively expressing ΔDCN in WAT. Plasmalemma-bound MMP14 is known to cleave its substrates before they are secreted from the cell [47]. We therefore compared the MMP14 expression level in ASC to other cells. To achieve that, we applied a *PDGFRb* lineage tracing strategy that we recently reported to identify adipocyte progenitors in mice [36]. By crossing the *Pdgfrb-Cre* and *mTmG* strains, we generated progeny in which mT (membrane Tomato) is indelibly replaced by mG (membrane GFP) fluorophore expression in *Pdgfrb+* ASC (Figure 1F). We then used FACS to separate mG+ (ASCs) and mT+ cells (endothelial cells, leukocytes, and *Pdgfrb-* ASCs) from the WAT of these mice (Figure 1G). Reverse transcription polymerase chain reaction (RT-PCR) revealed a five-fold higher expression of *MMP14* in *Pdgfrb+* ASC, compared to other cells in WAT (Figure 1G). This is consistent with ASCs being the cell population expressing DCN [30].

We also performed single cell RNA sequencing (scRNA-seq) to more precisely identify the ASC population in which MMP14 and DCN are co-expressed. We analyzed 4773 SVF cells isolated from subcutaneous WAT (SAT), which identified the same populations as the one previously reported [48,49,50]. Based on *Dpp4*, *Cd142*, and *Icam1* expression, we identified ASC and pre-adipocytes along with endothelial cells, and various leukocytes (Figure 1H). Expression of collagen chains *Col1a1* and *Col6a1* was observed mainly in ASC and preadipocytes (Figure 1H). Confirming lineage tracing data, expression of *Mmp14* and *Dcn* was concordant for ASC (Figure 1H).

Based on these data, we predicted that MMP14 should be co-localized with ΔDCN in ASC at the protein level. Because ΔDCN-specific antibodies are not available, we used peptide WAT7, binding to ASC-expressed ΔDCN, as a surrogate marker of ΔDCN expression [31]. Analysis of peritumoral stroma, adjacent to prostate lesions spontaneously developing in HiMyc mice [51], revealed WAT7 binding to MMP14-expressing cells (Figure S1E). WAT7 binding to ASC expressing MMP14 was also observed in AT surrounding grafts of HiMyc-derived HMVP2 cells in mice (Figure S1F). Combined, our results indicate that DCN is cleaved by MMP14 expressed in stromal cells prior to adipocyte differentiation, which explains why ΔDCN marks ASC.

### 3.2. ECM Defect in Skin and Adipose Tissue of Mice Lacking GAG of DCN

**Figure 2:**
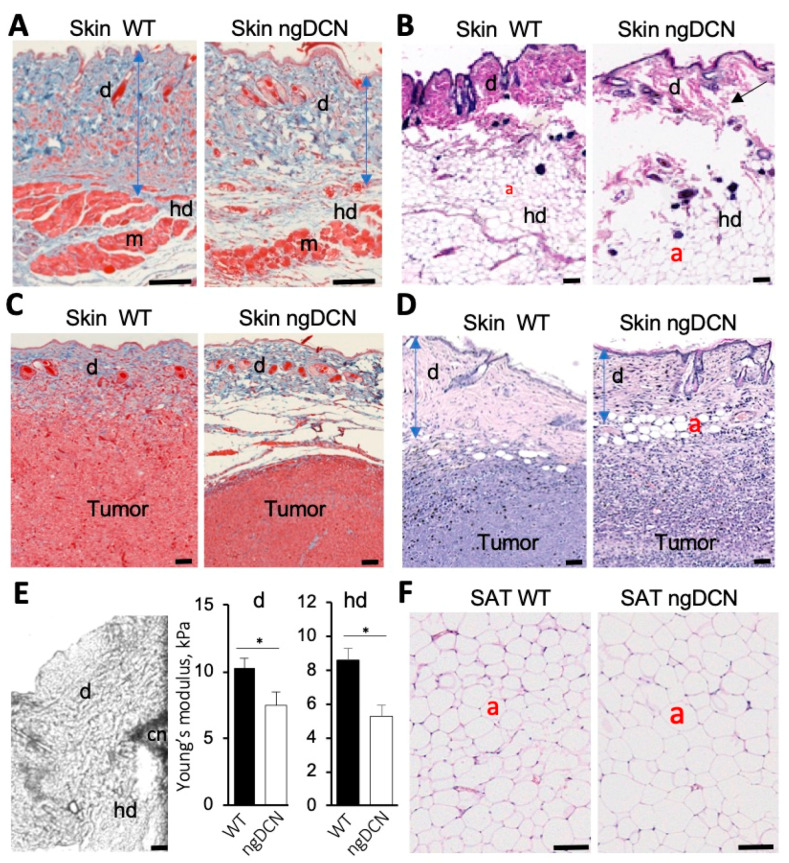
ECM defect and adipocyte enlargement in mice lacking GAG of DCN. (**A**,**B**) Sections of skin from WT and ngDCN knock-in (KI) mice stained with Masson’s trichrome (**A**) and H&E (**B**). Note the thinner dermis (d), its detachment from the hypodermis (hd), abnormal dermal myofibers (m), and larger dermal adipocytes (a) in ngDCN mice. (**C**,**D**) Sections of skin surrounding subcutaneous RM1 tumors grafted into WT and ngDCN knock-in mice stained with Masson’s trichrome (**C**) or H&E (**D**). Note the thin and loose dermis (d) and fibrous tumor capsule delamination in ngDCN mice. (**E**) Atomic force microscopy. Image: An example of the cantilever (cn) placed at the border of the dermis (d) and hypodermis (hd). Graphs: Mean Young’s modulus of the dermis and hypodermis measured for 50 locations per sample. N = 5 independent samples. Error bars = SEM; * *p* < 0.05 (Student’s *t*-test). (**F**) H&E-stained sections of SAT showing larger adipocytes sizes in ngDCN mice relative to WT mice. Scale bar = 50 µm.

To investigate the function of DCN GAG modification, we used a knock-in (KI) mouse strain with a point mutation in DCN disabling its o-glycosylation, as reported previously [23]. While electron microscopy demonstrated abnormal organization of collagen fibrils of the tendon and cornea in that study, no obvious phenotypic abnormalities were detected in these non-glycanated DCN KI (ngDCN) mice [23]. Here, we examined ngDCN mice, paying particular attention to the organs in which COL1 and COL6 dysfunction is detrimental. Sections of formalin-fixed skin revealed that ngDCN mice have a relatively thin and brittle dermis, as well as loose hypodermal connective tissue (Figure 2A and Figure S2A) and thinner dermal muscle fibers (Figure 2A). Because obesity often exacerbates genetic defect manifestations in WAT, we analyzed mice fed with a high-fat diet (HFD). In these mice, we observed larger adipocytes in dermal WAT (Figure 2B). Notably, splitting of the subdermal layers underneath the panniculus carnosus was observed (Figure 2B). At the age of 10 months, ngDCN mice have developed skin lesions visible macroscopically (Figure S2B). Both male and female ngDCN mice displayed the stretchy skin phenotype (Figure S2C). The skin defect was particularly obvious upon subcutaneous grafting of the tumors: skin peeled off from the tumors more easily in ngDCN mice. Histological analysis revealed fibrous tumor capsule delamination in ngDCN mice, which was not observed in WT littermates (Figure 2C). The dermal layer over the tumor was also thinner in ngDCN mice (Figure 2C,D and Figure S2D). In addition, there was a difference in matrix organization and increased leukocyte infiltration in internal areas of tumors grown in ngDCN mice (Figure 2C,D and Figure S2D). There was also a trend for faster tumor growth in ngDCN mice (Figure S2E).

To quantify matrix abnormalities in ngDCN mice, we used atomic force microscopy (AFM) on cryosections of mouse skin as described previously [42]. Nanomechanical measurements were obtained by microscopy-guided pressing of the cantilever into the dermis at 10 different locations per sample (Figure 2E). Young’s modulus was then estimated using a linear elastic based theory for a cone. The results revealed that both the dermis and hypodermis had a decreased elastic modulus in four-month-old ngDCN mice (Figure 2E). The reduced dermis and fat tissue stiffness accounts for the reduced tensile strength. These findings suggest that the GAG of DCN is required for normal ECM assembly.

### 3.3. The Effect of DCN GAG Absence on Adipose Tissue and Metabolism

Analysis of inguinal SAT sections revealed the presence of significantly larger adipocytes in ngDCN mice relative to WT mice (Figure 2F and Figure S3A). Comparable kinetics of lipid droplet formation was observed for cultured preadipocytes from ngDCN and WT mice induced for adipogenesis (Figure S3B). This result suggests that adipocyte hypertrophy is a result of ECM abnormality. Larger adipocyte lipid droplets were also observed in interscapular brown adipose tissue (BAT), an organ responsible for adaptive thermogenesis [52] (Figure S3C). There were no signs of overt steatosis in livers of ngDCN mice (Figure S3D). Lipid droplet hypertrophy BAT is often linked with metabolic alterations. However, cold tolerance test did not reveal a significant defect in thermogenic capacity of the ngDCN mice (Figure S3E). Indirect calorimetry measurements in metabolic chambers also did not reveal a defect in oxygen consumption (VO_2_), an indicator of energy expenditure (Figure S3F). Food consumption was also comparable in WT and ngDCN mice (Figure S3G). These data indicate that, despite an ECM defect, ngDCN mice are protected from metabolic dysfunction typically arising in conditions linked with adipocyte hypertrophy.

### 3.4. ECM Defect in Skeletal Muscle of Mice Lacking GAG of DCN

**Figure 3:**
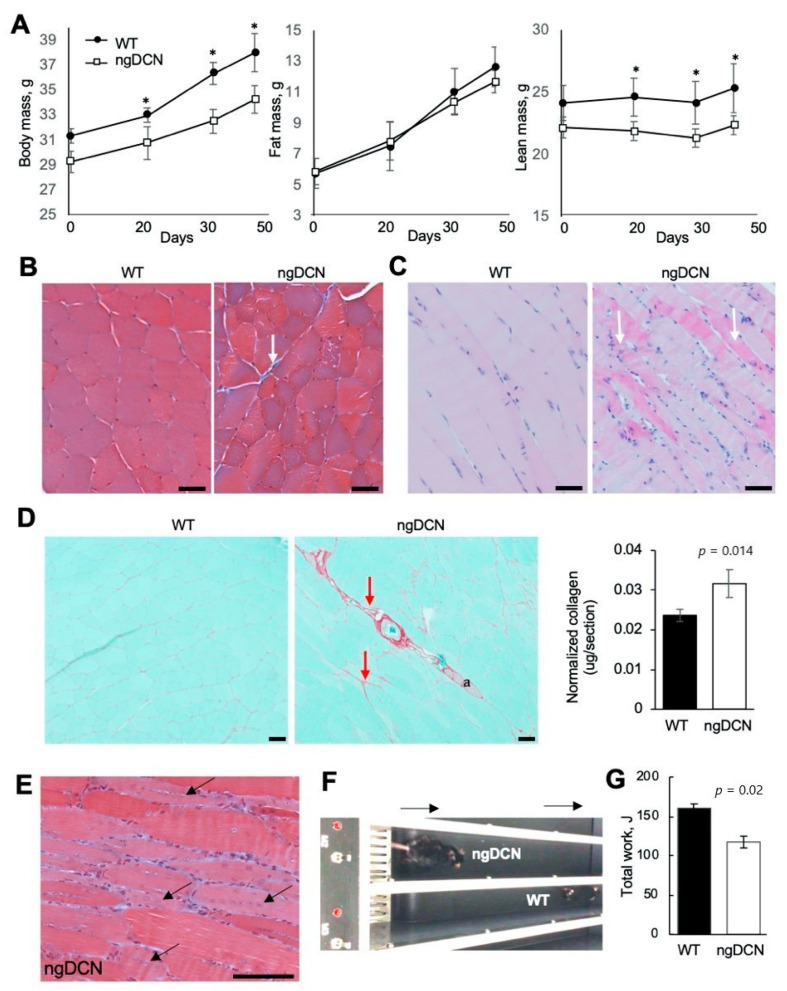
Skeletal muscle abnormality and weakness in mice lacking GAG of DCN. (**A**) Echo MRI data reveals WT-comparable fat mass and lower lean mass of ngDCN mice placed on HFD for 45 days. N = 6 mice per group. * *p* < 0.05 (Student’s *t*-test) compared to WT mice. (**B**) Cross-section of gastrocnemius muscle stained with Masson’s trichrome reveal fiber size irregularity and fibrosis (arrow) in ngDCN mice. (**C**) Longitudinal sections of gastrocnemius stained with H&E reveal increased cellularity and fiber basophilia (arrows) in ngDCN mice. (**D**) Picrosirius red staining with fibrotic lesions highlighted (arrows) in cross-sectioned gastrocnemius of ngDCN mice. Graph: Red collagen signal normalized to total green protein. N = 5 sections per group. (**E**) Longitudinal section of ngDCN gastrocnemius demonstrates central nuclei (arrow), indicating fiber regeneration. (**F**) A snapshot of a WT and ngDCN littermate running on a treadmill (arrows: direction), illustrating reduced fatigue resistance capacity in ngDCN mice. (**G**) Quantification of data from (**F**) from 10 runs of N = 5 WT and N = 5 ngDCN mice, showing reduced physical endurance of ngDCN mice reflected in Joules of work performed. Scale bar = 50 µm.

Despite HFD feeding, ngDCN mice were found to lag behind WT littermates in body weight (Figure 3A). This was surprising, provided the adipocyte hypertrophy observed in ngDCN mice (Figure 2C). Body composition analysis revealed that, while fat mass has increased normally in ngDCN mice, it is their lean mass that has become significantly lower than in WT mice with age (Figure 3A). Because muscle accounts for the bulk lean body mass, we analyzed hind limb skeletal muscle. Masson’s trichrome staining of the gastrocnemius cross-sections revealed heterogeneity of muscle fiber thickness and prominent fibrotic lesions in ngDCN mice (Figure 3B–D). Longitudinal sections revealed signs of muscle remodeling and a highly increased cellularity in ngDCN mice (Figure 3C). There was increased presence of basophilic myofibers (Figure 3C), which is characteristic of chronic muscle regeneration in muscular dystrophy due to protein synthesis and the expression of developmental forms of MyHC [53]. Consistent with that, centrally localized nuclei, indicative of satellite cell recruitment for myofiber regeneration [53], were observed in myofibers of ngDCN mice (Figure 3E). To assess functional implications of this phenotype, we subjected mice to running on a treadmill. Compared to WT littermates, ngDCN mice displayed a significantly reduced fatigue resistance, as shown in a representative video (Supplementary Material), its snapshot (Figure 3F), and quantification of the total work (Figure 3G). Combined, these observations suggest that matrix abnormality resulting from the lack of DCN GAG compromises skeletal muscle regeneration and function.

### 3.5. Collagen Level Abnormality in Organs of Mice Lacking GAG of DCN

**Figure 4:**
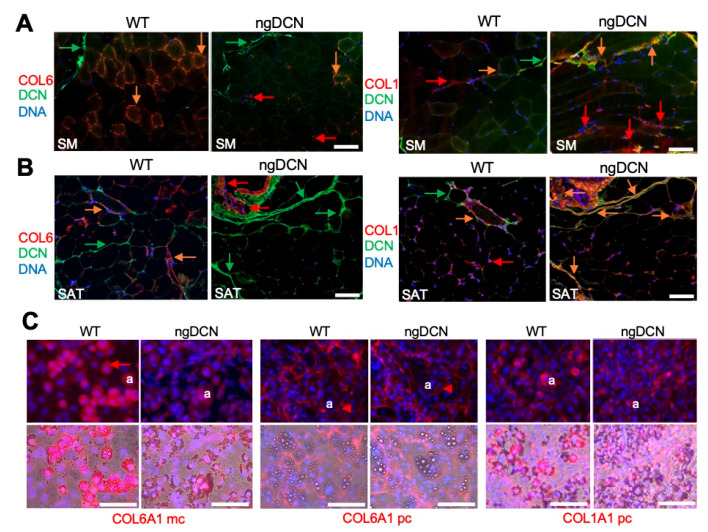
Collagen expression abnormality in skeletal muscle and WAT of ngDCN mice. (**A**,**B**) IF with anti-DCN (green) and anti-COL6A1 or anti-COL1A1 (red) antibodies on sections of skeletal muscle (SM, (**A**)) and SAT (**B**) from WT and ngDCN mice. Colocalization of DCN and collagen is indicated with orange arrows. Note the decreased COL6A1/DCN co-localization in SM and SAT of ngDCN mice. (**C**) Cultured MEFs from WT and ngDCN mice differentiated into adipocytes and subjected to IF (red) with anti-COL6A1 monoclonal (mc), anti-COL6A1 polyclonal (pc), or anti-COL1A1 antibodies. For ngDCN, COL6A1 in adipocytes (a) is decreased intracellularly (arrow) but is comparable extracellularly (arrowhead). Brightfield-merged images show lipid droplets. Similar results were obtained from the analysis of N = 5 independent view fields per group. Nuclei are blue. Scale bar = 50 µm.

To investigate the cause of the matrix defect in ngDCN mice, we analyzed collagen expression in tissue sections by IF with a monoclonal antibody specific for the COL6A1 chain and polyclonal antibodies against COL1A1. In WT mice, COL6A1 was highly expressed in skeletal muscle in co-localization with DCN around most myofibers (Figure 4A). In contrast, the COL6A1 IF signal was lower in myofibers of ngDCN mice and mainly localized at fibrotic lesions free of DCN (Figure 4A). The COL1A1 IF signal was also abundant in fibrotic lesions of skeletal muscle from ngDCN mice, where it partly co-localized with DCN (Figure 4A). Similarly, in SAT, a lower COL61A signal was observed in adipocytes of ngDCN mice, compared to WT mice (Figure 4B and Figure S4A). As in muscle, the COL61A signal in ngDCN SAT was mainly observed in fibrotic lesions free of DCN (Figure 4B and Figure S4A). COL1A1 was also abundant in fibrotic lesions of SAT from ngDCN mice, where it co-localized with DCN (Figure 4B and Figure S4A). Notably, DCN deposition appeared to be more pronounced in SAT of ngDCN mice (Figure 4B).

To establish the cause of the collagen distribution changes, we analyzed MEFs differentiated into adipocytes in cell culture. IF with the monoclonal anti-COL6A1 antibody, which mainly recognized intracellular COL6A1, revealed that its expression in WT adipocytes was higher than in ngDCN adipocytes (Figure 4C). However, IF with polyclonal anti-COL6A1 antibodies, which preferentially stained extracellular collagen, did not reveal obvious differences in pericellular COL6A1 levels (Figure 4C). The adipocyte COL1A1 IF signal was also not markedly different in WT and ngDCN adipocyte cultures. However, picrosirius red staining revealed an abundance of fibrotic lesions in SAT of ngDCN mice (Figure S4B). Combined, these results suggested that collagen chain processing and localization in the matrix is abnormal in the absence of DCN GAG.

### 3.6. Defective Collagen Production in Adipocytes of Mice Lacking GAG of DCN

**Figure 5:**
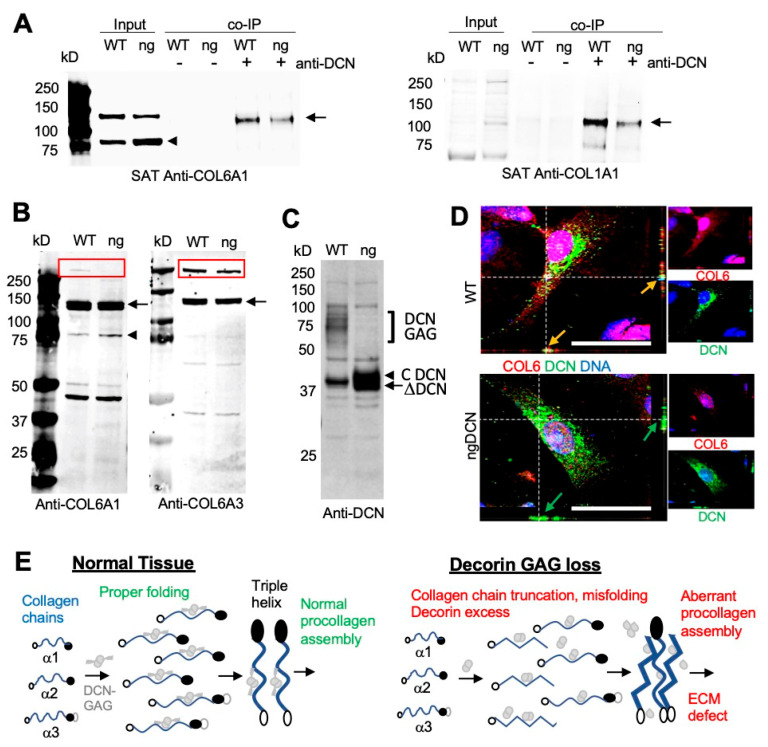
Abnormal DCN binding and processing of collagen chains in ngDCN mice. (**A**) Anti-COL6A1 and anti-COL1A1 immunoblots of the protein extracts (Input) from SAT reveal a reduced level of full-length COL6A1 (arrow) and increased level of truncated COL6A1 (arrowhead) in ngDCN (ng) mice. Co-immunoprecipitation (co-IP) with anti-DCN IgG demonstrate less COL6A1 and COL1A1 chains (arrow) complexed with DCN in ngDCN mice. (**B**) Anti-COL6A1 and anti-COL6A3 immunoblots of protein extracts from WT and ngDCN (ng) adipocytes differentiated in cell culture after removal of ECM proteins. Note the increased formation of a truncated COL6A1 fragment (arrowhead), reduced levels of COL6 chains (arrow), and reduced levels of COL6 procollagen (box) migrating at ~500 kDa. (**C**) Anti-DCN immunoblots of the protein extracts reveal the lack of glycanated DCN and increased levels of non-glycanated DCN in lysates of ngDCN MEFs differentiated into adipocytes. (**D**) Confocal immunolocalization of DCN and COL6A1 in cultured primary ASC. Z-stack projections of median series reveal the change in COL6A1 distribution and reduced DCN colocalization in ngDCN cells. Scale bar = 50 µm. (**E**) A model depicting the role of DCN GAG in collagen fibrillogenesis. The glycanated DCN dimer binds to the three collagen VI chains and organizes their correct folding for proper procollagen formation. Lack of GAG on DCN results in increased DCN expression and collagen chain C-terminal truncation, which leads to elevated assembly of aberrantly folded procollagen and fibrosis.

To further investigate collagen expression, we analyzed tissue protein extracts by Western blotting. From denaturing gel analysis, the levels of COL6A1 and COLA1 chains appeared to be comparable in WT and ngDCN muscle and WAT (Figure S4C). Native gels revealed the presence of expected COL6A1 monomers, dimers, and trimers in ngDCN WAT (Figure S4D). The monoclonal antibody against the N-terminus of COL6A1 revealed a ~80 kDa protein fragment in both WT and ngDCN extracts (Figure 5A). This truncated chain was not detected with the polyclonal COL6A1 antibody (Figure S4C), indicating that it recognizes a C-terminal epitope. While the 140 kDa band, corresponding to the full-length chain, was more abundant than the 80 kDa truncation band in WT mice, the opposite was observed for ngDCN (ng) extracts (Figure 5A). This indicated that the absence of DCN glycan jeopardizes COL6A1 processing. To specifically look at collagens interacting with DCN, we performed immunoprecipitation (IP) with DCN antibodies from WAT protein extracts. Probing the pulldowns with anti-collagen antibodies revealed that both COL6A1 and COL1A1 are in complex with DCN in WAT. However, lower amounts of COL6A1 and COL1A1 chains were pulled down from the WAT extract of ngDCN mice than of the WT mouse extract (Figure 5A), which is consistent with reduced DCN/collagen co-localization observed in ngDCN mice (Figure 4A,B).

We also investigated whether the defect in collagen processing occurs during its intracellular synthesis. For this, we analyzed adipocytes differentiated from ASC in cell culture. To enrich for intracellular collagens prior to secretion into the ECM, we thoroughly washed the trypsin-detached cells off the extracellular proteins prior to cell lysis and protein extraction. Immunoblotting demonstrated that the reduction in full-length COL6A1 chain and the increase in the 80 kDa fragment occurs prior to secretion in ngDCN adipocytes (Figure 5B). To test if this is chain-specific, we used an antibody specific for endotrophin, a C-terminal fragment of COL6A3 [38]. Immunoblotting also revealed a reduction in the amount of full-length COL6A3 chain in ngDCN adipocytes and confirmed that the cleavage product is the N-terminal part of COL63A not recognized by the endotrophin antibody (Figure 5B). While a ~500 kDa COL6 procollagen monomer band assembled from the three chains was observed for WT, a reduction in its intracellular level was detected for ngDCN adipocytes with both COL6A1 and COL6A3 antibodies (Figure 5B).

Immunoblotting of protein extracts revealed a marked higher level of non-glycanated DCN in extracts from cultured ngDCN adipocytes, compared to WT adipocytes (Figure 5C). However, IF analysis of primary mouse WT and ng DCN ASC did not reveal a drastic difference in intracellular DCN levels (Figure 5D). This suggests that the excess DCN is mainly extracellular in vivo, which is consistent with our SAT analysis (Figure 4B). IF on ASC demonstrated high expression of COL6A1 throughout the cell body and its co-localization with DCN in WT ASC (Figure 5D). In contrast, in ngDCN cells, the COL6A1 signal was reduced to puncta, which was more prominent in the cytosol than on the cell surface (Figure 5D). Combined, our data indicate that ngDCN, which has a lower collagen affinity than GAG-DCN, increases C-terminal truncation of the COL6 chains, hence steering improper collagen chain assembly and localization (Figure 5E).

## 4. Discussion

DCN is a matricellular proteoglycan mediating assembly of collagens I and VI as well as the interactions between these collagens [12,54]. DCN plays multiple roles in soft tissue physiology and cancer progression [12,55]. We previously discovered that ΔDCN, a proteolytic DCN isoform lacking the N-terminus containing the GAG attachment site, is generated in WAT [30]. Here, we show that ΔDCN is a product of cleavage by metalloprotease MMP14 in a series of in vitro and cell culture studies, as well as by using transgenic mice overexpressing MMP14 in adipocytes. By using knock-in (KI) mice expressing only non-glycanated ngDCN (ngDCN), we show that GAG of DCN is required for normal dermis and hypodermis organization and skin rigidity. Subcutaneous tumor growth was slightly increased and peritumoral inflammatory infiltrate was more pronounced in ngDCN mice. We show that ngDCN mice have enlarged adipocytes and display signs of myopathy and muscular dystrophy in their skeletal muscle. We provide evidence that this phenotype is due to abnormal collagen deposition in WAT and muscle tissues. Finally, we demonstrate decreased intracellular DCN-collagen binding, decreased intracellular procollagen levels, and increased generation of truncated COL6 chains in ngDCN mice. Based on these results, we propose a working model according to which, in the absence of DCN glycan, the ECM accumulates more abnormal collagen fibers dissociated from DCN, which results in increased fibrosis (Figure 5E).

The role of O-glycan (GAG) modification on DCN has remained controversial because ngDCN mice had been reported to lack an overt phenotype, although collagen fiber irregularity was apparent from that study in younger animals [23]. However, the lack of O-glycan modification on DCN is observed in patients with Ehlers–Danlos syndrome [21], manifestations of which we now find recapitulated in ngDCN mice. These features, including skin fragility, SAT overgrowth, and fatigue associated with muscle weakness, also hallmark mutations in collagens I and VI. Importantly, a progressive loss in DCN O-glycanation is also observed in aging, also marked by skin fragility, adipocyte enlargement, and sarcopenia [22]. These hallmarks of aging are linked with obesity, a condition associated with type 2 diabetes development [56,57,58]. The metabolic syndrome in obese patients typically results from adipocyte hypertrophy and hypoxia, leading to fibrosis and inflammation [59,60]. The obesity pathogenesis involves dysregulated secretion of collagens as well as of DCN [24,59]. Notably, while mice lacking COL6 expression in WAT have hypertrophic adipocytes, they are metabolically healthy due to adipocytes being less constrained by the ECM [61]. It remains to be determined how DCN deglycanation is affected by obesity and what the long-term repercussions of this condition are during aging and upon obesity development. The potentially distinct roles of chondroitin and dermatan sulfate modification also remain to be addressed.

MMP14 mediates collagenolytic processes [8] and, hence, plays an important role in ECM remodeling [33,62]. MMP14 dysfunction is linked with aging-associated pathologies and MMP14-deficient mice display fibrosis of soft tissues [63]. Conversely, mice induced for adipocyte overexpression of MMP14, cleaving DCN and collagen, display a healthy metabolic profile despite adipocyte hypertrophy [7], consistent with the phenotype of ngDCN mice. As we reported, this phenotype flips in older MMP14-overexpressing mice, which ultimately develop WAT fibrosis and become diabetic upon prolonged HFD feeding [7]. This indicates that extreme adipocyte enlargement eventually results in cell death inflammation and fibrosis irrespective of initial ECM permissiveness. It has been previously reported that DCN is cleaved by MMP14 [43]. However, the function of MMP14 to generate ΔDCN in WAT has not been reported. Because DCN, as well as MMP14, are expressed by adipocytes, we have hypothesized that ΔDCN is produced extracellularly and is deposited on the ASC surface from the outside [30]. However, here we show that DCN and MMP14 are co-expressed in ASC prior to differentiation. This provides an explanation of how ΔDCN is generated in ASC. While cell surface-localized MMP14 cleaves many ECM substrates, intracellular cleavage by MMP14 has also been demonstrated [47,64]. The exact sub-cellular location where DCN cleavage by MMP14 occurs remains to be determined. MMP14 is highly expressed in WAT, in particular in obesity [34], and regulates WAT remodeling [65]. MMP14 is also highly expressed in solid cancers and mediates tumor invasion [66,67]. Our studies have reported that MMP14 cleaves the COL6A3 chain to generate endotrophin, a bioactive peptide associated with obesity pathogenesis [38,68,69]. The relative importance of DCN, collagen VI, and other substrates as the MMP14 targets underlying ECM remodeling in WAT and skeletal muscle remains to be determined. Collagen VI, DCN, and MMP14 are also functional players in the tumor microenvironment [70,71]. As confirmed in this study, stromal cells with ΔDCN on the surface reside in tumors surrounded by WAT [31]. It remains to be determined if the function of cancer-associated fibroblasts [72] relies on ΔDCN. It is possible that MMP14 at the invasive tumor front plays a dual role in cancer progression by deglycanating DCN and remodeling ECM to enable cancer cell invasion. However, because MMP14 induces the activity of other proteases, including MMP2 [7], the relative functional importance of various direct and indirect MMP14 targets remains to be elucidated.

The mechanisms through which the GAG of DCN could mitigate matricellular interactions may be multi-pronged and mediated by interactions with molecules other than collagens [12]. We previously established that ΔDCN, expressed on the ASC surface, serves as a receptor for an inflammation-associated cytokine resistin [30]. As we reported previously, expression of ΔDCN increased the proliferation and migration of stromal cells while suppressing their adipogenic capacity [30], indicating its role as a signaling molecule. How DCN deglycanation affects its interaction with TGF-β and other growth factors remains to be determined. However, our study indicates the important role that GAG of DCN plays in collagen interaction. A reduction in procollagen production observed in cells of ngDCN mice may appear to be at odds with the increased muscle and AT fibrosis observed for that model. However, mutations in *COL6A1* and other *COL6* chain genes also result in congenital muscular dystrophies and are similarly characterized by increased interstitial fibrosis and adipogenesis [73]. A phenotype strikingly similar to that of ngDCN, both in the skin and skeletal muscle, has been observed for mouse *Col6a1* mutants [4,74,75]. In our study, a reduction in intracellular COL6A1 levels was observed in skeletal muscle and SAT of ngDCN mice. Notably, this was detected with a monoclonal antibody binding to its N-terminus. Immunoblotting with antibodies selective for the N- or C-termini of collagens revealed a reduction in the full-length COL6 chains and increased levels of a truncated N-terminal 80 kDa COL6A1 fragment in ngDCN adipocytes. In the future, identifying the mechanism of collagen chain truncation in the absence of DCN GAG will be important. Previous studies investigating the role of DCN GAG at early steps of collagen fibrillogenesis demonstrated that non-glycanated DCN is more potent in increasing collagen fibril diameter [17]. Consistent with our observations, it has been reported that core DCN has a lower affinity for collagens I and VI than glycanated DCN [76]. While DCN is known to mediate interactions between collagens I and VI [54,76], it has been shown that GAG is not required for inter-collagen binding [77]. New tools to accurately measure properly folded and functional collagen chains and their multimerization and interaction with other ECM molecules, including DCN, may be necessary to establish the nuances of DCN involvement in collagen fiber assembly and extracellular localization.

## 5. Conclusions

In summary, our study indicates that DCN lacking GAG is generated by cleavage with MMP14 and suggests that MMP14 induction in obesity is responsible for the accumulation of DCN lacking GAG. It also points to the pathogenic role of GAG-less DCN, which compromises normal collagen synthesis and localization, leading to matrix dysfunction typical of aging and genetic collagen defects. Site-specific cleavage of ECM and of matricellular proteins by proteases, resulting in new bioactive byproducts, is emerging as a fundamental mechanism of cell-matrix signaling. Systematic discovery of proteolytic protein isoforms will help to better understand the mechanisms regulating connective tissue function.

## Supplementary Materials

The following are available online at https://www.mdpi.com/2073-4409/9/12/2646/s1. Figure S1 ΔDCN in cells and mouse tissues.; Figure S2 Subcutaneous tumor matrix defect in mice lacking GAG of DCN.; Figure S3 Adipogenesis and metabolism in ngDCN mice.; Figure S4 Collagen matrix abnormality in ngDCN mice.
