# Supplementary material for: Glycosaminoglycan Modification of Decorin Depends on MMP14 Activity and Regulates Collagen Assembly
Source: Cells. 2020 Dec 9;9(12):2646. doi: 10.3390/cells9122646 (PMC7764107; doi:10.3390/cells9122646)
Supplement: Supplementary file 1 [file cells-09-02646-s001.pdf]

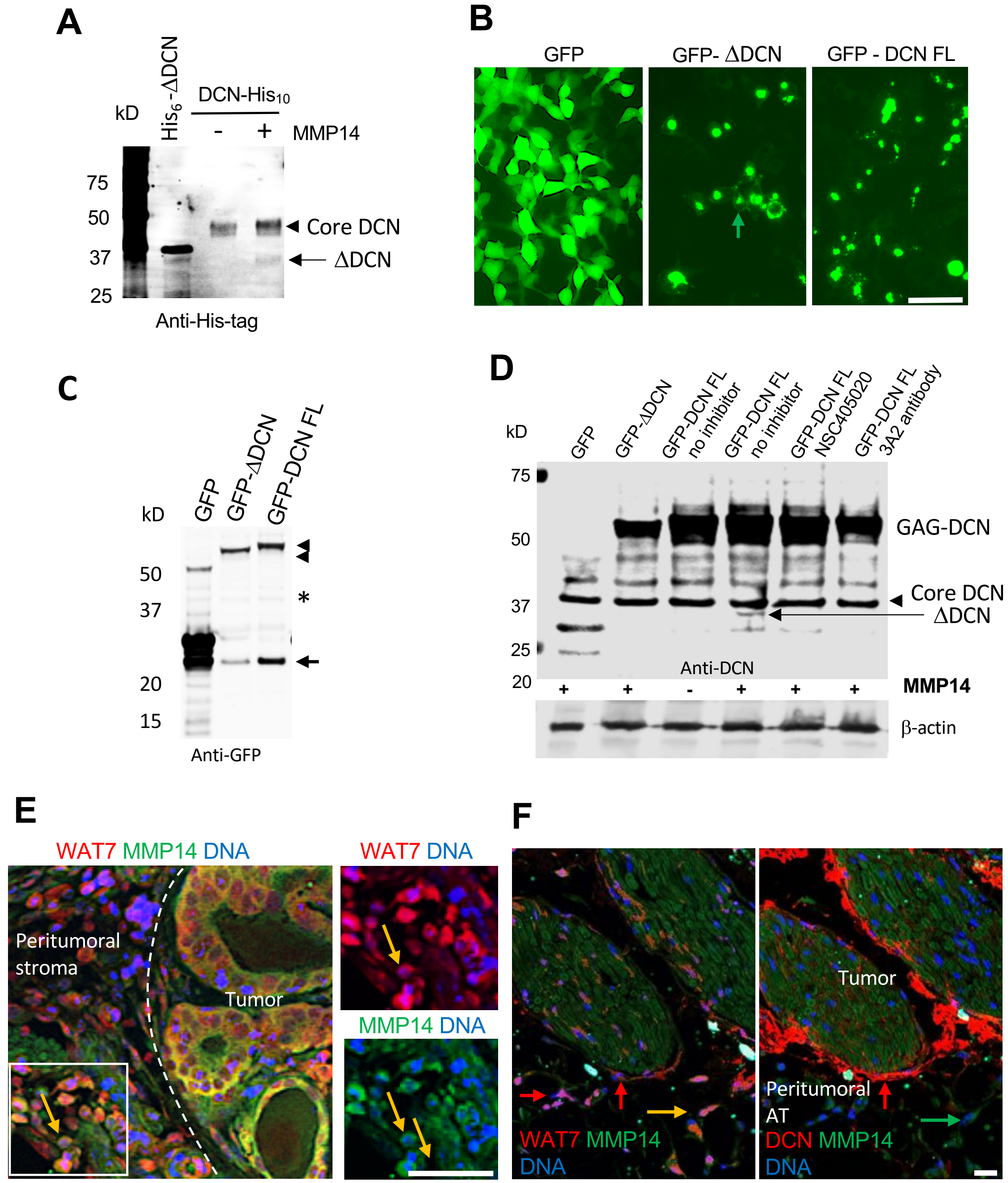

Fig. S1 ΔDCN in cells and mouse tissues. **A**, Protein extracts from Fig. 1B immunoblotted and probed with anti-His antibody (Rockland #200-303-382) confirming that the cleavage fragment has the His Tag and is hence C-terminal. **B**, Expression of GFP fusions shown in Fig. 1C visualized by GFP fluorescence in HEK293 cells. Note the surface localization of GFP-ΔDCN (arrow). **C**, Anti-GFP immunoblotting of extracts from HEK293 co-transfected with MMP14 and GFP fusions (Fig. 1D) reveals increased cleavage releasing N-terminal GFP (arrow) in cells expressing GFP-DCN FL compared to GFP-ΔDCN. Note that GFP expression is much higher without DCN fused. Non-cleaved GFP-DCN-FL and GFP-ΔDCN bands (arrowheads) and nonspecific bands (*) indicate comparable protein loading. **D**, Anti-DCN immunoblotting of extracts from HEK293 cells expressing the indicated GFP fusions and co-transfected with MMP14 as indicated. Note that pre-treatment of MMP14-expressing cells with MMP14 inhibitor NSC405020 (50 μM) and a blocking antibody 3A2 (1 μg/ml) suppresses generation of ΔDCN (arrow). Endogenous core DCN and GAG-DCN are present in all HEK293 extracts. High expression of free GFP results in additional nonspecific bands. Actin immunoblot: loading control. **E**, IF on tumors from HiMyc mice showing MMP14 expression by WAT7-bound (ΔDCN-expressing) cells at the invasive front (dashed line). **F**, Serial sections of HMVP2 tumor grafts showing WAT7 binding to ASC expressing MMP14 (yellow/green arrow) in peritumoral AT. Full length DCN is mainly deposited in the matrix surrounding lesions (red arrow). Scale bar=50 μm.

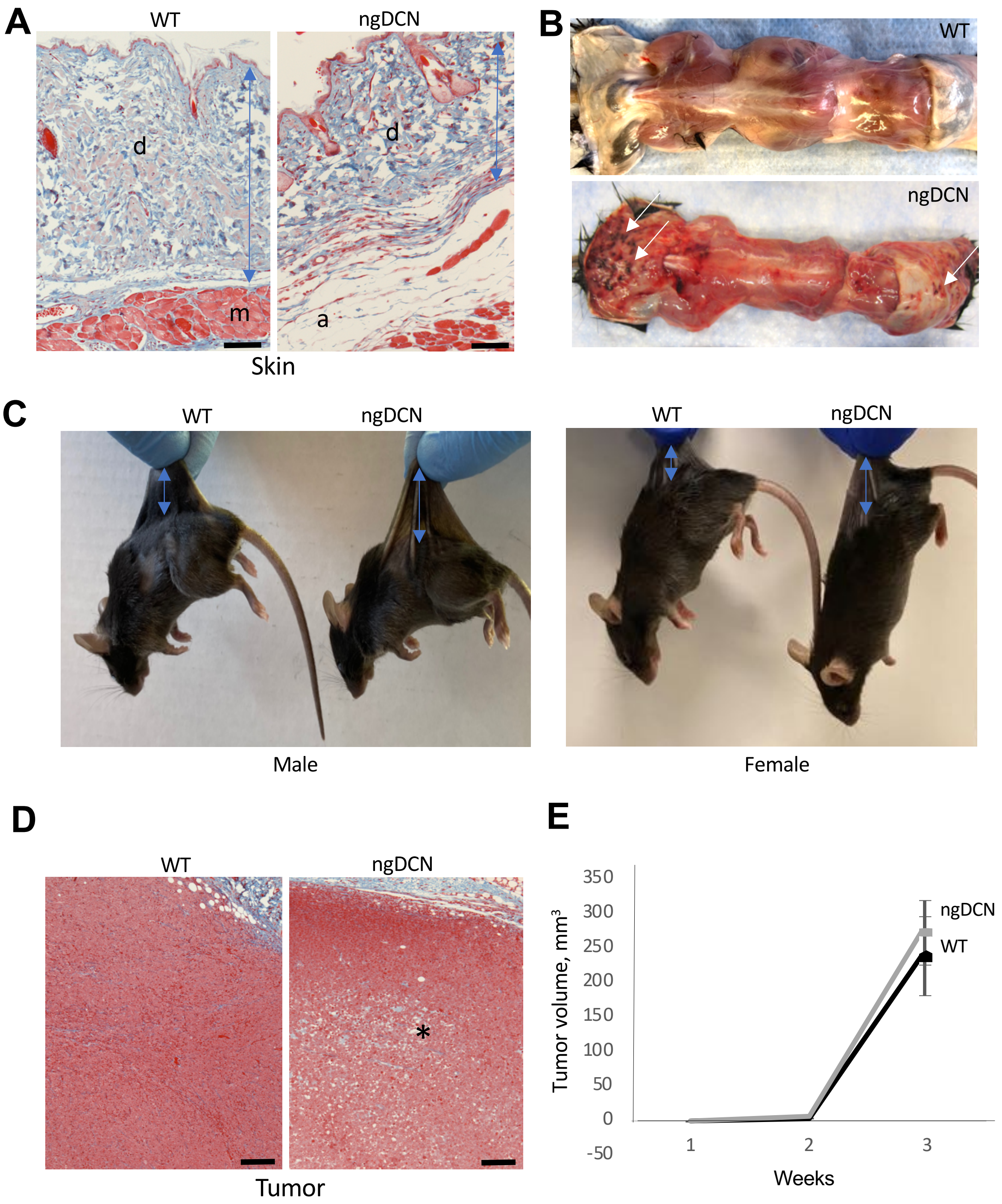

Fig. S2 Subcutaneous tumor matrix defect in mice lacking GAG of DCN.
A, Sections of skin showing thinner dermis (arrow), increased hypodermal adiposity (a) and reduced myofiber (m) thickness in ngDCN mice. **B**, Internal skin lesions (arrows) observed in 10-month-old ngDCN mice but not in WT mice. **C**, Demonstration of increased skin stretch (arrows) in pinch-hung male and female ngDCN mice. **D**, Sections of subcutaneous RM1 tumors showing a change in intratumoral matrix (*) of ngDCN mice. **E**, RM1 graft tumor growth rate in WT and ngDCN mice (N=10). Scale bar=50 μm. Error bars: SEM.

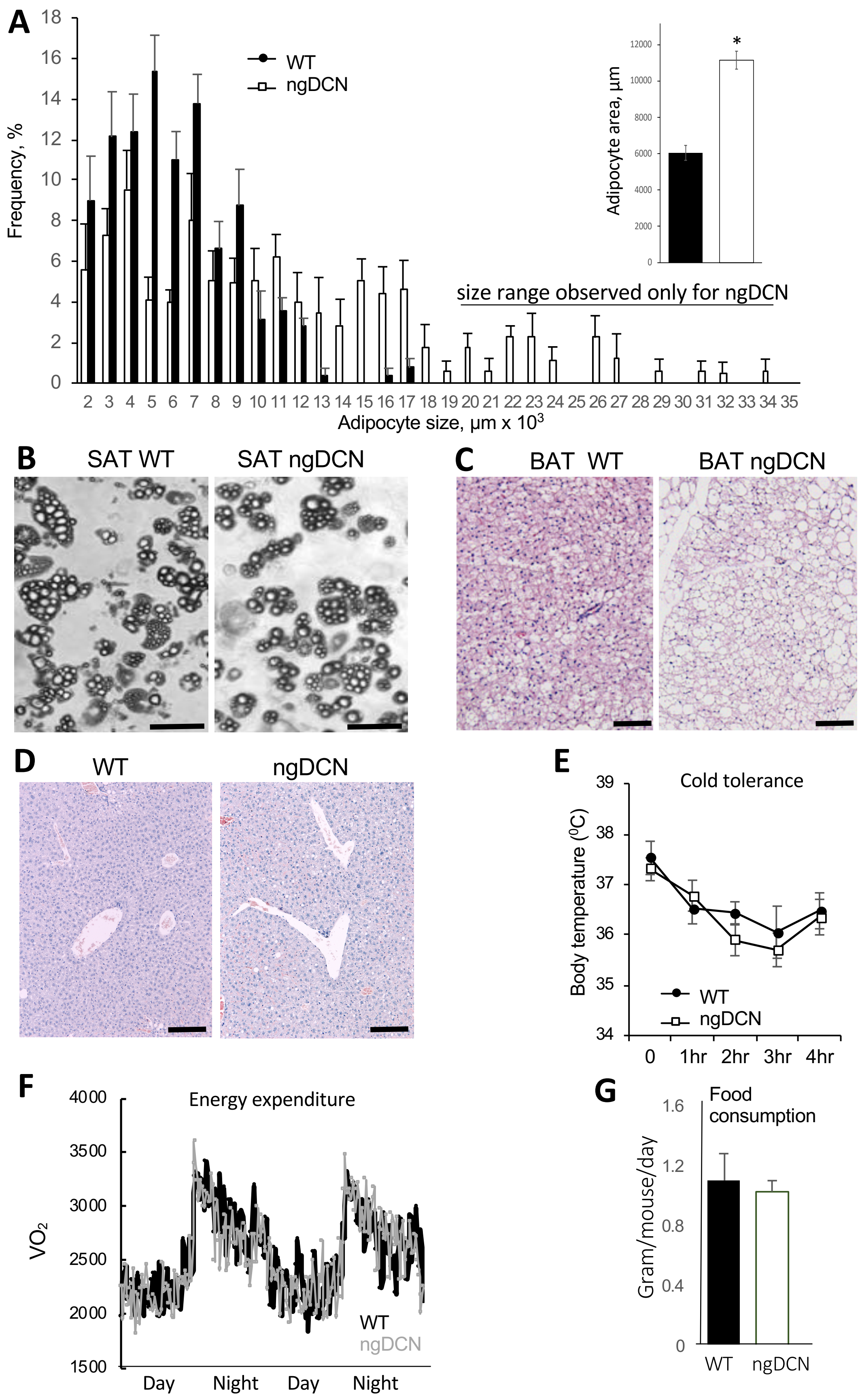

Fig S3 Adipogenesis and metabolism in ngDCN mice. N = 6 mice per group. **A**, Adipocyte size frequency (left) and average area (right) quantified using Adiposoft plugin of Image J software using N=5 independent H&E staining section images of SAT. **B**, ASC from SAT of WT and ngDCN mice subjected to adipogenesis *ex vivo* reveal comparable lipid droplet formation. **C**, H&E-stained sections of interscapular BAT showing larger lipid droplets in brown adipocytes of ngDCN mice. **D**, Sections of mouse livers stained with H&E. **E**, Core body temperature measured over 4 hr at 4°C reveals comparable cold tolerance in WT and ngDCN mice. **F**, Indirect calorimetry reveals comparable oxygen consumption (VO_2) in WT and ngDCN mice. **G**, Food consumption measured over 3 days is comparable in WT and ngDCN mice. Scale bar=50 µm. Error bars: SEM.

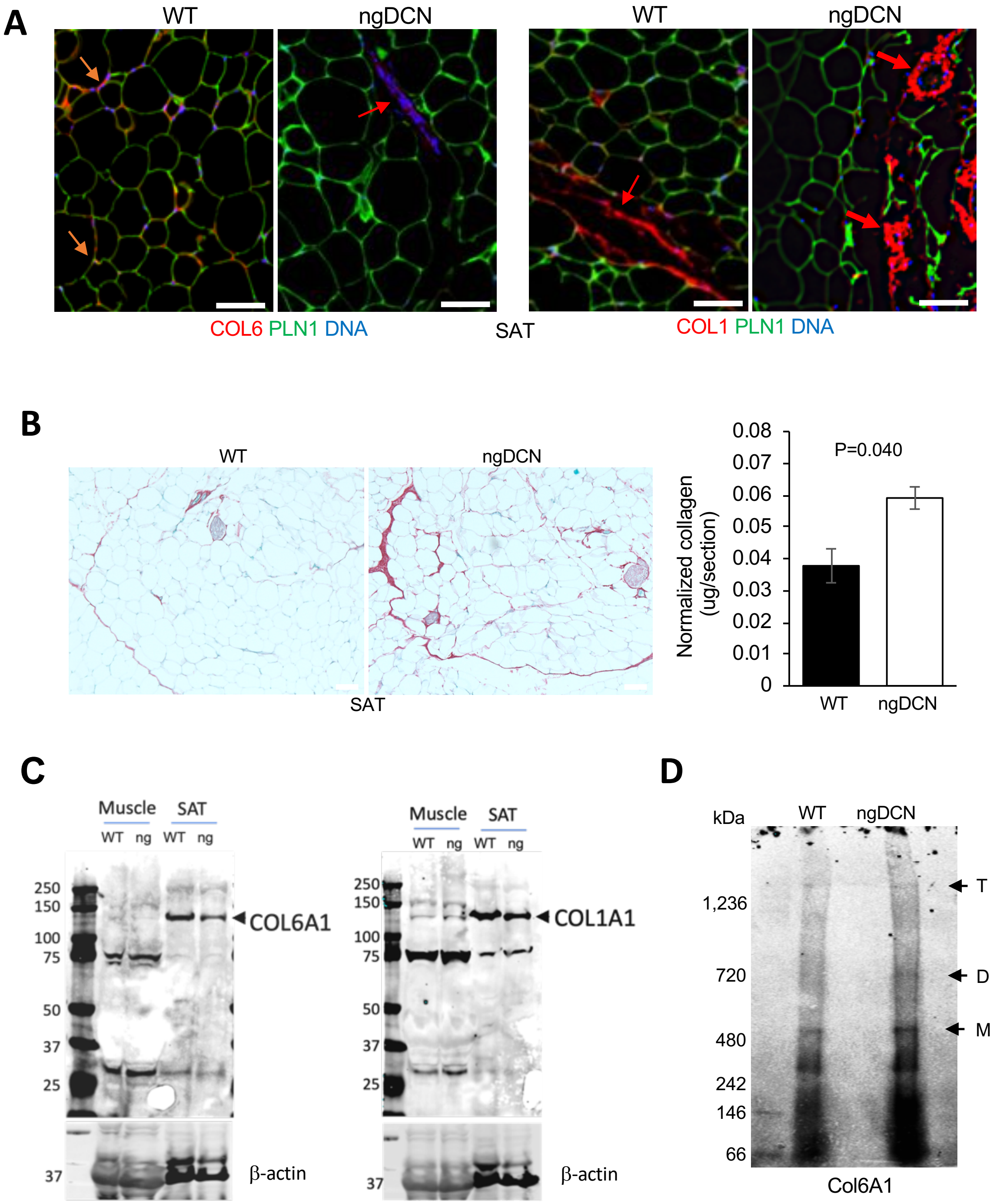

Fig S4 Collagen matrix abnormality in ngDCN mice. **A**, IF of SAT from WT and ngDCN KI mice with antibodies against perilipin-1 (PLN1), COL6A1 and COL1A1. Colocalization of PLN1 and collagen in adipocytes is indicated with orange arrows. Fibrotic lesions are indicated with red arrows. **B**, Picrosirius red staining highlighting fibrotic lesions in cross-sectioned SAT. Graph: red collagen signal normalized to total green protein. N=5 sections. **C**, Anti-COL6A1 (ab6588 polyclonal) and anti-COL1A1 (polyclonal) immunoblots of protein extracts from SAT of WT and ngDCN (ng) mice resolved on denaturing gel. **D**, 3-12% native gel electrophoresis of WT and ngDCN SAT extracts followed by immunoblotting with anti-COL6A1 antibody. Arrows indicate the bands matching the expected migration of triple-helix monomers (M, ~500 kDa), disulfide-bonded antiparallel dimers (D, ~1,000 kDa), and tetramers (T, ~2000 kDa) observed in both WT and ngDCN mice. Scale bar=50 µm. Error bars: SEM.
